# Differential cellular localization of lectins in the testes of dromedary camel (*Camelus dromedarius*) during active and inactive breeding seasons

**DOI:** 10.1186/s12917-023-03791-1

**Published:** 2023-11-04

**Authors:** Mahmoud S. Gewaily, Mohamed Gaber Abdallah, Norhan E. Khalifa, Ola A. Habotta, Ahmed E. Noreldin

**Affiliations:** 1 Department of Anatomy and Embryology, Faculty of Veterinary Medicine, Kafrelsheikh University, Kafrelsheikh, 33516 Egypt; 2 Department of Medical Biochemistry, Faculty of Medicine, Al-Azhar University, Cairo, Egypt; 3 Department of Physiology, Faculty of Veterinary Medicine, Matrouh University, Matrouh, 51511 Egypt; 4 Department of Forensic Medicine and Toxicology, Faculty of Veterinary Medicine, Mansoura University, Mansoura, 35516 Egypt; 5 Department of Histology and Cytology, Faculty of Veterinary Medicine, Damanhour University, Damanhour, 22511 Egypt

**Keywords:** Dromedary camel, Testes, Lectins, LHC, Spermatogenesis

## Abstract

The reproductive activity of the male dromedary camel (*Camelus dromedarius*) as a seasonal breeder is affected by various seasonal changes that reflect on the reproductive performance. In the current study, we explored a differential cellular localization of lectins in eight dromedary camel testes utilizing lectin histochemistry (LHC). The glycoconjugates’ localizations were detected within the testicular tissue utilizing 13 biotin-labeled lectins (PNA, ConA, LCA, RCA120, GS IB4, WGA, BPL, DBA, ECA, PHA-E4, UEA-1, PTL-II, and SBA) distributed into six sets. The cellular structures revealed diverse lectins distribution that may reflect various glycoproteins’ structures and their compositional modifications during spermatogenesis. Some of the investigated lectins were restricted to acrosomes of spermatids that will help study different stages during the spermatogenic cycle of dromedary camel, particularly PNA, and ECA. The statistical analysis showed a marked positive correlation between the response intensity of various lectins and the breeding season (P < 0.05). We can conclude that lectins have a fundamental role during camel spermatogenesis and are associated with the reproductive activity of dromedary camel.

## Introduction

Camel has a high ability to survive under harsh conditions [1, 2]. The reproductive activity of camels was a main subject that was interesting to many researchers [3–5]. It is well known that reproduction in camel is affected by many various factors affecting spermatogenesis including hormonal, biochemical, cytokines, and direct interaction between somatic and spermatogenic cells. All these factors are in turn affected by seasonal variations. During the rutting or breeding period, the male camel reveals higher sexual activity [6–8]. The dromedary camel as a seasonal breeder has extreme gross, microscopic, and ultra-structural morphological changes in genital organs, which give rise to different hormonal urges, which in turn lead to different reproductive activities and sexual behaviors during different seasons [9].

The camel testis showed seasonal and monthly alterations but there is no complete activity stoppage [3, 10]. During all seasons, spermatogenesis is persistent but it is significantly activated through the rut season [7, 9, 11–18]. Camel testes includes several glycoconjugates (containing glucosyl, galactosyl, and mannosyl residues), and they are deficient in fucosyl residues, both in the active breeding season and in the non-breeding season [3].

Spermatogenesis is a highly self-renewing productive mechanism that consumes around 30 − 75 days in various mammals [19, 20]. Although the spermatogenesis features are similar in all mammals [21], some characteristics such as morphological characteristics of germ cells, number of generations, and type can vary between species [22, 23]. Cellular glycoconjugates depend on interactions between carbohydrates and proteins indicating the oligosaccharides importance [24, 25]. The sugar codes express the biological information and the glycan epitopes transmission [26–30].

The lectins are non-immune glycoproteins that precipitate glycoconjugates [31–34]. Lectins could be termed as non-immunoglobulin proteins able to specifically recognize and reversibly bind to carbohydrate moieties without changing the covalent composition of the glycosyl ligands [33, 35]. Lectins could be obtained from invertebrates, plants, or vertebrates, and they have specific affinities for sugar sequences or glycoconjugate sugar residues [36, 37]. Therefore, lectins have been utilized to detect the distribution of glycoconjugate in malignant and normal tissues [38–40]. Furthermore, many reports have confirmed that cellular glycoconjugates have pivotal functions such as motility, development, and growth regulation [41–43]. Many studies have revealed various modulations in the sugar residues within the spermatocytes that differ between species such as some domesticated mammals [30, 44–46], rodents [47, 48], and humans [49].

It is well known that most previous studies were concerned with the evaluation of either testicular morphology or the hormonal profile of camel. In addition, the previous studies on the lectins in the dromedary testes were very few. So, the aim of this work referred to elucidate the seasonal variations of a wide range of sugar residues in the dromedary camel testis at the level of LHC and their association with reproductive performance to provide helpful data for better understanding spermatogenesis occurred during active and inactive breeding seasons.

## Materials and methods

### Animal samples

Eight male apparently healthy adult camels were used in the present study. The samples were obtained from Kom Hamada abattoir (four camels in the Summer and Winter seasons). The right testis was taken just after slaughtering. The specimens were gathered in 4% paraformaldehyde solution in phosphate buffer saline (PBS) for lectin histochemistry.

### Lectin histochemistry (LHC)

LHC was conducted according to MS Gewaily, M Kassab, A Aboelnour, EA Almadaly and AE Noreldin [30]. Briefly, the sections were deparaffinized and rehydrated till saturated with distilled water. Then, the antigen retrieval was applied using 0.02 M of Tris HCL (PH 9) autoclaved for 15 min at 95ºC. After washing with PBS, the non-specific reactions were discarded utilizing 1% bovine serum albumin (Sigma-Aldrich) in PBS solution including 0.05% NaN3 for 5 min. We incubated sections with biotin-labeled lectins (1:200, Vector Laboratories, Burlingame, CA) (Table 1) at room temperature for 1 h. After washing with PBS, the immunoreaction was visualized by Streptavidin, Alexa Fluor™ 594 conjugate (1:400, Invitrogen) incubated for 1 h at room temperature pursued by PBS washing. The sections were counterstained by Hoechst 33,258 (1:1000, Sigma-Aldrich) at room temperature for 10 min. After PBS washing, we mounted sections utilizing Fluoromount™ (Diagnostic BioSystems, Japan). Finally, the examination of the stained sections was carried out by fluorescence microscope (BZ-X710; Keyence, Japan).

**Table 1:** Biotinylated lectins that have been used in this study, sugars binding specificities, and their inhibitory sugars. Man: mannose. Glc: glucose, Fuc: fucose, Gal: Galactose, GlcNAc: N-acetylglucosamine, GalNAc: N-acetyl galactosamine

Lectin group	Name	Source	Specificity	Concentration µ/ml	Inhibitory sugar
Glucose- binding lectins	LCA	Lens culinaris	α-Man	5	Man
	ConA	Concanavalin A	α-D- Man, α-D-Glc	10	Man
Galactose -binding lectin	PNA	Peanut agglutinin	Gal β1-3GalNAc	20	Gal
	RCA 120	Ricinus communis 120	Gal β1-4GlcNAC	5	Gal
	GS-IB4	Griffonia simplicifoIia IB4	D-Gal	5	Gal
Glucosamine- binding lectin	WGA	Wheat germ agglutinin	β-D-GlcNAc	5	GlcNAc
Galactosamine -binding lectins	BPL	Bauhinia purpurea Lectin	GlcNAc	20	GalNAc
	DBA	Dolicos biflorus	GalNAc α 1–3 GalNAc	20	GalNAc
	ECA	Erythrina Crista galli Agglutinin	Gal, GalNAc,	20	GalNAc
	SBA	Soybean Agglutinin	GalNAc	20	GalNAc
	PTL-II	Psophocarpus tetragonolobus	GalNAc	20	GalNAc
Fucose -binding lectin	UEA-1	Ulex europaeus − 1	α-L-Fuc	20	Fuc
Nonspecific lectins	PHA-E4	Phaseolus vulgaris	D-GalNAc	5	GalNAc

Specific reactions of lectin staining were confirmed by exposure of negative control sections to Streptavidin without lectins. The intensities of staining were grouped into five classes: no labeling, weak labeling, moderate labeling, strong labeling, and very strong labeling as (–), (+), (++), (+++), and (++++) respectively. The biotinylated lectins utilized in this investigation and their inhibitory sugars and binding specificities are revealed in Table 1.

### Statistical analysis

We classified the reactions of each cell to various lectins depending on the staining intensities. 1 (negative response), 2 (low response), 3 (medium response), 4 (strong response), and 5 (very strong response). We studied the correlation between the response intensity and several variables, such as lectin type, lectin class, breeding season, and testicular cell type utilizing a generalized linear regression model (ordinal logistic regression) with the response intensity as the output variable [50]. We assessed the odds ratios to evaluate the correlation between the variable of response intensity and other variables. We carried out the statistical analyses utilizing IBM SPSS Statistics for Windows, Version 23.0 (Armonk, NY. USA).

## Results

**Fig. 1:**
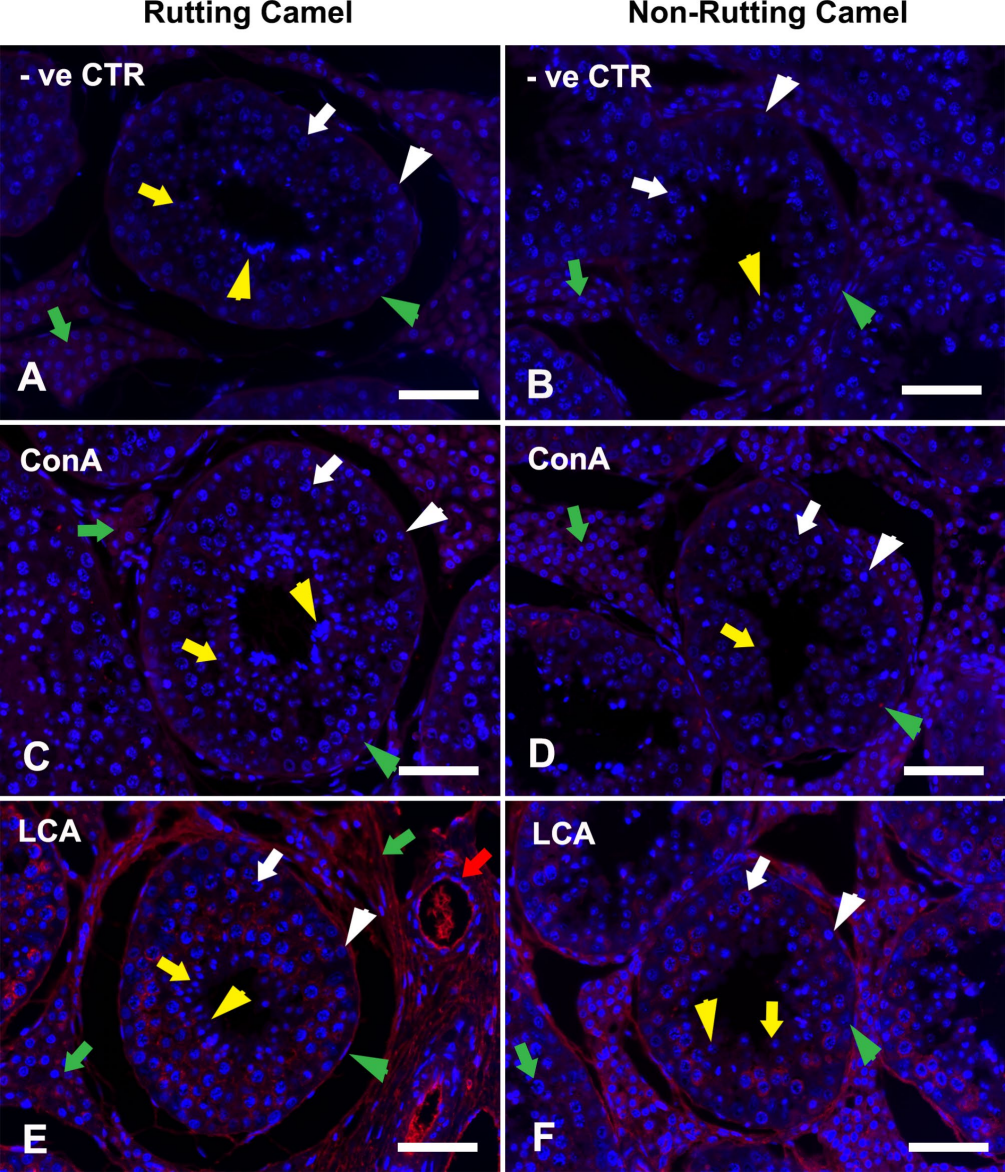
lectin histochemistry (LHC) in the testes of dromedary camel revealing positions of glucose (Glc) binding lectins; ConA, and LCA as well as a negative control during rutting **(A, C, E)** and non-rutting **(B, D, F)** seasons. The cellular nuclei were counterstained with Hoechst 33,258 (blue fluorescence). Spermatogonia; Sg: white arrowhead, Spermatocyte; Sp: white arrow, round spermatid; Rs: yellow arrow, elongated spermatid; Es: yellow arrowhead, Sertoli cell: green arrowhead, and interstitial cells; IC: green arrow. There was not any reactivity in the negative control section in both breeding and non-breeding seasons. ConA was mildly labeled in spermatocytes and interstitial cells in both seasons. LCA was more labeled in spermatogonia, spermatocytes, and interstitial cells but in a higher intensity (red fluorescence) in breeding than in the non-breeding season in addition to the basement membrane and interstitial blood vessels (red arrow). Scale bar = 100 μm

All investigated lectins in the present study showed different labeling intensities in the camel testicular cells either spermatogenic (spermatogonia; Sg, spermatocytes; Sp, round spermatids; Rs, and elongated spermatids; Es), somatic Sertoli cells or interstitial cells (IC). This localization pattern was confirmed by the negative control result (Fig. 1A and B). The labeling intensities of different lectins are shown in Table 2.

**Table 2:** The labeling intensities of different lectins in the one-humped camel testis during rutting and non-rutting seasons. The used lectin groups included Glc; Glucose binding lectin, Gal; Galactose binding lectin, GlcNAc; Glucose amine binding lectin, GalNAc; Galactose amine binding lectin, Fu; Fucose binding lectin, and NS; Nonspecific binding lectin. Sg; spermatogonia, Sp; spermatocytes, Rs; round spermatids, Es; elongated spermatids, Sc; Sertoli Cells, IC; interstitial Cells. The levels of labeling were described as negative (-), weak (+), moderate (++), strong (+++), and very strong (++++)

Lectin group	Name	Rutting camel testes						Non-Rutting camel testes					
		**Sg**	**Sp**	**Rs**	**Es**	**Sc**	**IC**	**Sg**	**Sp**	**Rs**	**Es**	**Sc**	**IC**
Glc	**ConA**	-	+	-	-	-	+	-	+	-	-	-	+
	**LCA**	++	++++	-	-	-	++++	+	+++	-	-	-	+++
Gal	**PNA**	++	+	+++	++++	-	-	+	-	++	+++	-	-
	**RCA120**	++	++	++	++++	-	++++	-	-	+	+++	-	++
	**GS-IB4**	+++	++	-	+++	-	++	-	+	-	+	-	++
GlcNAc	**WGA**	+	++++	++	+++	-	-	+	++++	++	++	-	-
GalNAc	**BPL**	-	+	++++	++++	-	+	-	+	++	++	-	+
	**DBA**	-	-	-	+	-	-	-	-	-	-	-	-
	**ECA**	+	-	++++	++++	-	-	-	-	++++	++++	-	-
	**SBA**	**+++**	**+++**	**+++**	+++	++	+++	++	++	++	+++	++	+
	**PTL-II**	-	+	++++	+++	-	+++	-	-	+	+	-	++
Fu	**UEA-1**	-	+	++	++	-	++	-	+	-	-	-	+
NS	**PHA-E4**	-	+++	+++	+++	-	+++	-	+++	+++	+++	-	+++

### Glucose (glc) binding lectins

**Fig. 2:**
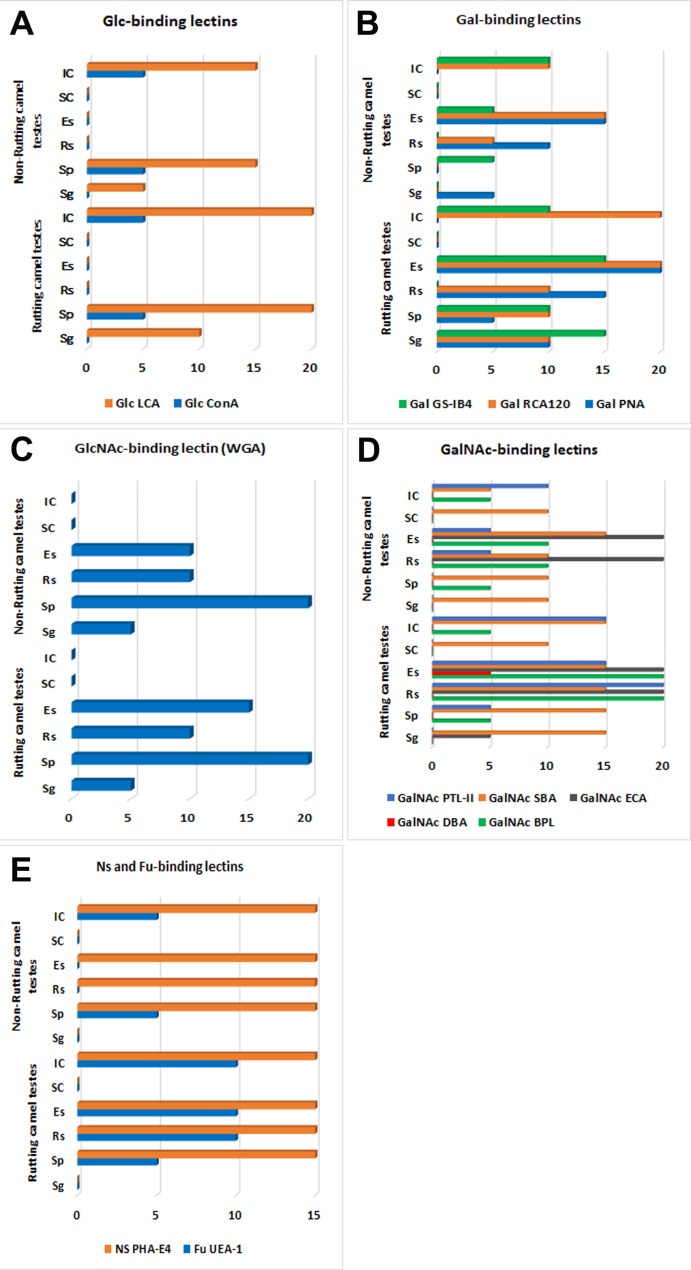
Summarized data representing the labeling intensities of different investigated lectins in the dromedary camel testes in different testicular cells during rutting and non-rutting season **(A-E)** spermatogonia (Sg), spermatocyte (Sp), round spermatid (Rs), elongated spermatid (Es), Sertoli cells (Sc) and interstitial cells (IC)

The Glc-binding lectins (ConA, LCA) were absent in Sertoli cells, round, and elongated spermatids in both breeding and non-breeding seasons. However, they were localized in other spermatogenic (Sg and Sp) and interstitial cells, they showed higher expression in rutting than non-rutting months. In addition, the basement membrane of the seminiferous tubule as well as interstitial blood vessels were strongly labeled by LCA (Figs. 1C-F and 2A).

### Galactose (gal) binding lectins

**Fig. 3:**
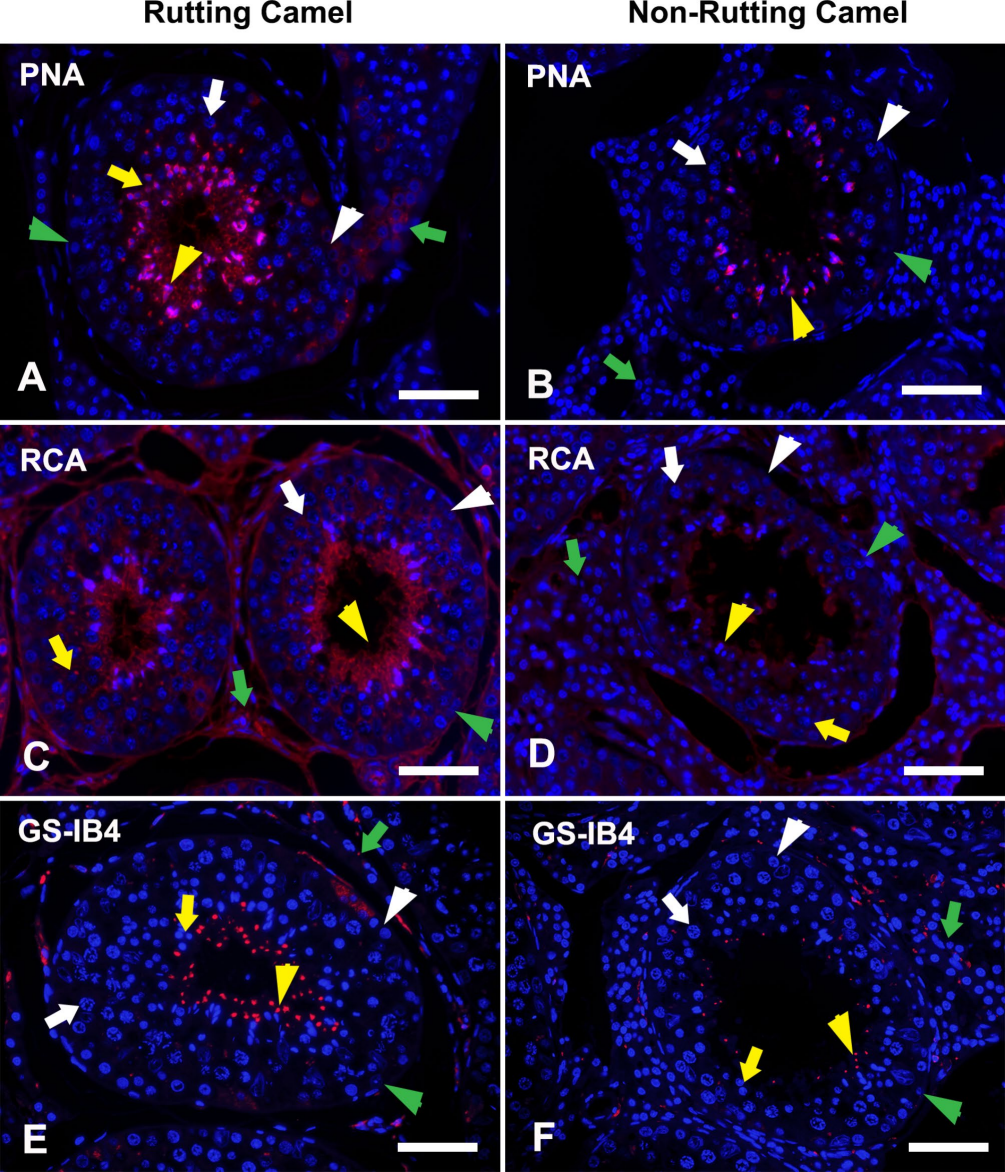
LHC in the testes of dromedary camel displaying positions of galactose (Gal) binding lectins; PNA (upper panel), RCA (middle panel), and GS-IB4 (lower panel) during rutting **(A, C, E)** and non-rutting **(B, D, F)** seasons. The cellular nuclei were counterstained with Hoechst 33,258 (blue fluorescence). Spermatogonia; Sg: white arrowhead, Spermatocyte; Sp: white arrow, round spermatid; Rs: yellow arrow, elongated spermatid; Es: yellow arrowhead, Sertoli cell: green arrowhead, and interstitial cells; IC: green arrow. The round and elongated spermatids clearly reacted to all investigated lectins within this group; however, the intensity (red fluorescence) was stronger in active than non-active seasons as well as interstitial tissue for RCA. Scale bar = 100 μm

Gal-binding lectin was examined via three lectins: PNA, RCA, and GS-IB4 which were not localized in Sertoli cells. The IC was not labeled by PNA and was moderately labeled by GS-IB4 all over the year. On the other hand, RCA labeling was more expressed in breeding than in non-breeding season. Both spermatogonia and spermatocytes were strongly labeled by Gal-binding lectins in rutting months or not labeled during non-rutting months. Notably, the round and elongated spermatids clearly localized all investigated lectins within this group; however, the intensity was stronger in active than non-active seasons (Figs. 3 and 2B).

### Glucose amine (GlcNAc) binding lectin

**Fig. 4:**
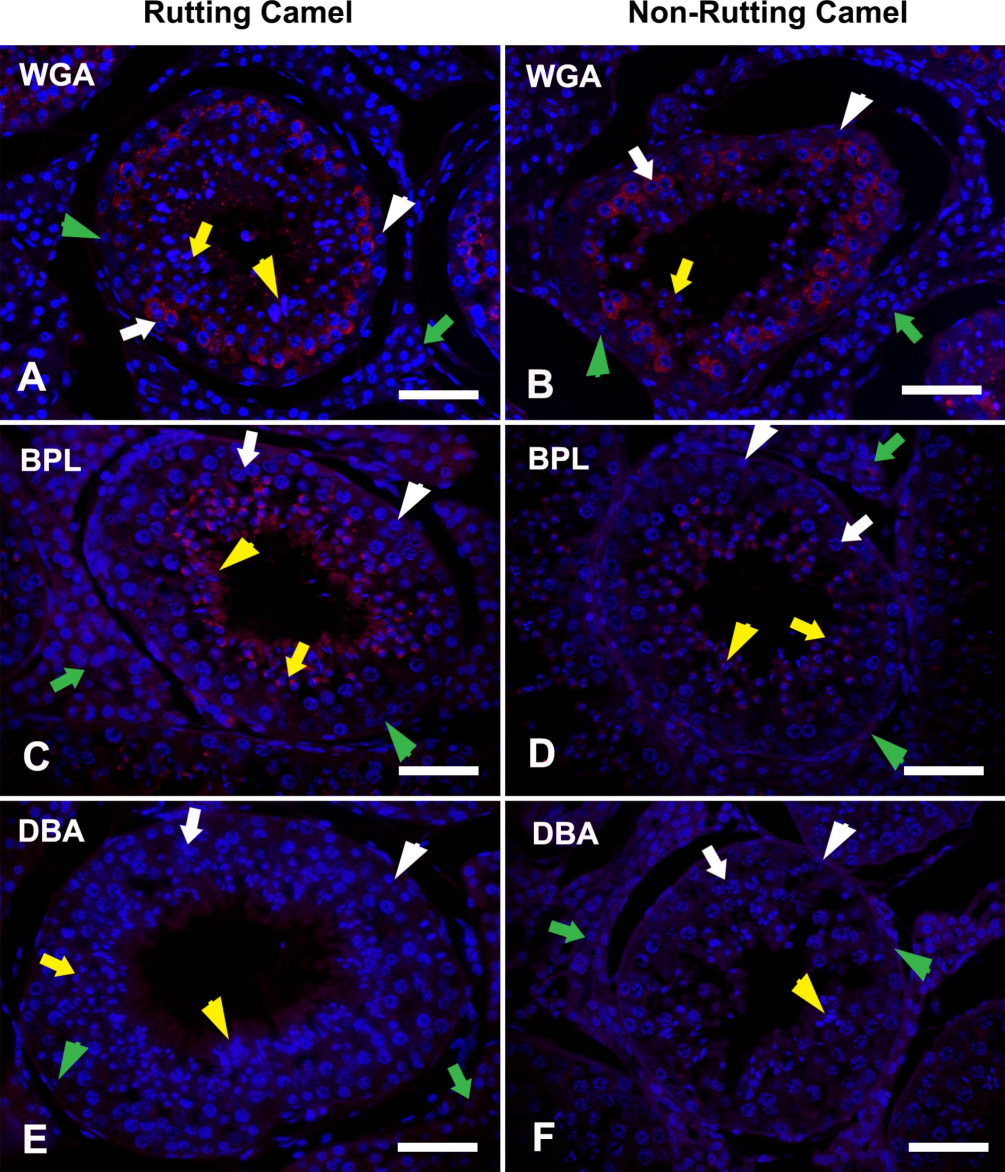
LHC in the testes of dromedary camel displaying positions of GlcNAc; WGA (upper panel) and GalNAc; BPL and DBA (middle and lower panels respectively) during rutting **(A, C, E)** and non-rutting **(B, D, F)** seasons. The cellular nuclei were counterstained with Hoechst 33,258 (blue fluorescence). Spermatogonia; Sg: white arrowhead, Spermatocyte; Sp: white arrow, round spermatid; Rs: yellow arrow, elongated spermatid; Es: yellow arrowhead, Sertoli cell: green arrowhead, and interstitial cells; IC: green arrow. WGA had nearly the same labeling pattern (red fluorescence) in both rutting and non-rutting camels’ testes. BPL was mainly restricted in the spermatids (Rs and Es) with strong expression during the rutting season. DBA was absent in most testicular cells except for weak localization in the elongated spermatid during the active breeding season. Scale bar = 100 μm

GlcNAc was investigated by WGA. It was worth notably that this lectin had nearly the same labeling pattern in both rutting and non-rutting camels’ testis. The WGA labeling affinity was restricted to spermatogonia (weak intensity), spermatocytes (very strong intensity), round spermatids (moderate intensity), and elongated spermatids (strong intensity). The other cellular component of the camel testis (Sertoli cells, IC) showed negative localization for WGA (Figs. 4A-B and 2C).

### Galactose amine (GalNAc) binding lectins

**Fig. 5:**
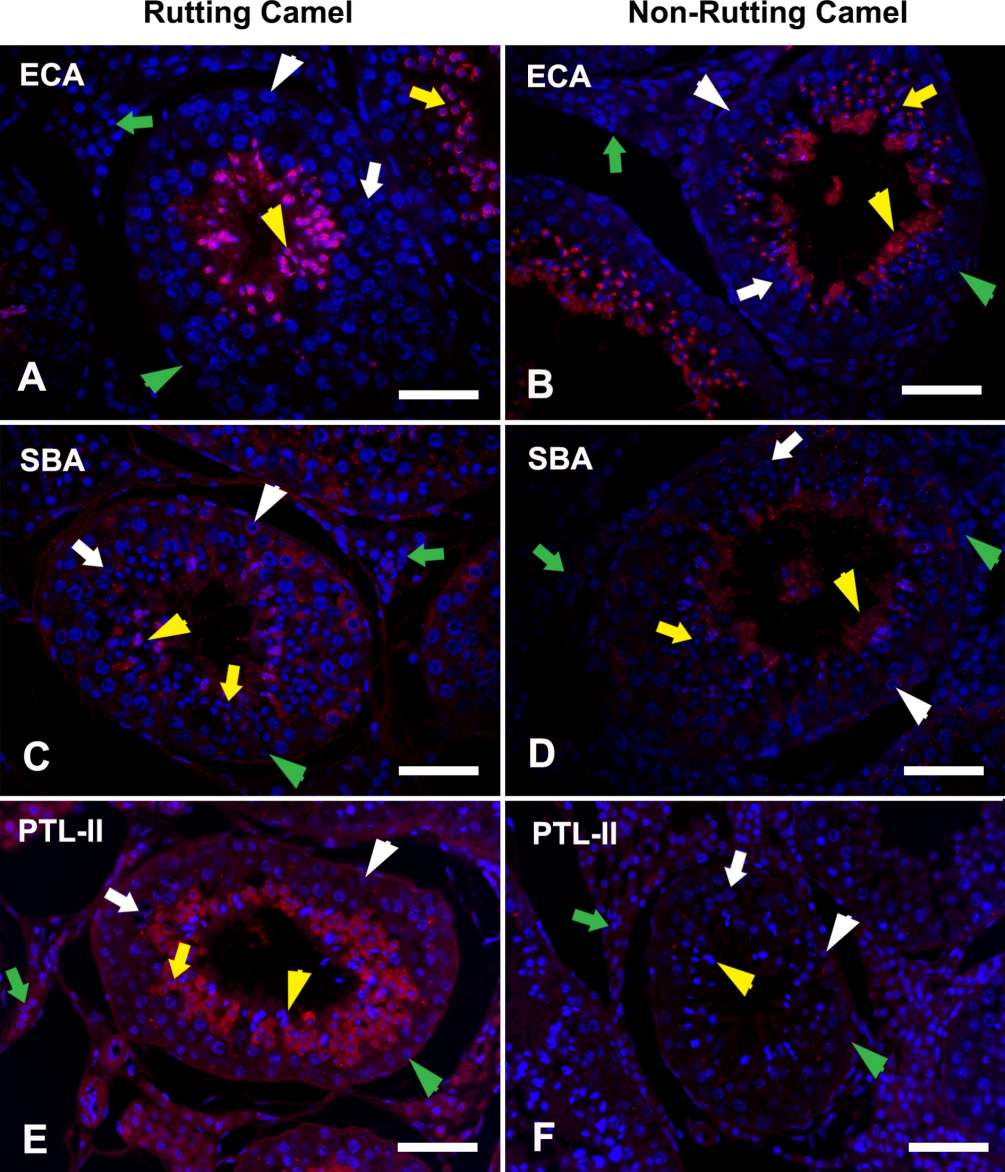
LHC in the testes of dromedary camel revealing positions of GalNAc; ECA (upper panel), SBA (middle panel), and PTL-II (lower panel) during rutting **(A, C, E)** and non-rutting **(B, D, F)** seasons. The cellular nuclei were counterstained with Hoechst 33,258 (blue fluorescence). Spermatogonia; Sg: white arrowhead, Spermatocyte; Sp: white arrow, round spermatid; Rs: yellow arrow, elongated spermatid; Es: yellow arrowhead, Sertoli cell: green arrowhead, and interstitial cells; IC: green arrow. ECA was restricted to the Rs and Es in a strong reactivity (red fluorescence) during both seasons. SBA was localized in all testicular cells even in the Sertoli cells but by the stronger intensity in rutting than non-rutting periods. PTL-II was strongly localized in rutting camel spermatids and IC while in the non-rutting camel, it was moderately found in IC and weak in the spermatids. Scale bar = 100 μm

GalNAc was examined in the testicular components of the camel testis by many lectins including BPL, DBA, ECA, SBA, and PTL-II. BPL was mainly restricted in the spermatids (round and elongated) with strong expression during the rutting season and moderate intensity during the inactive season in addition to weak localization in IC during both seasons (Fig. 4C-D). DBA was absent in most testicular cells except for weak localization in the elongated spermatid during the active breeding season (Fig. 4E-F). ECA was also restricted in the spermatids but in a strong labeling intensity during both seasons (Fig. 5A-B). SBA was localized in all testicular cells even in the Sertoli cells but by the stronger intensity in rutting than non-rutting periods (Fig. 5C-D). PTL-II was strongly localized in rutting camel spermatids and IC while in the non-rutting camel, it was moderately found in IC and weak in the spermatids while, it was absent in other testicular cells (Fig. 5E-F). The differential expression within this group of lectins was summarized in Fig. 2D.

### Fucose and nonspecific binding lectins

**Fig. 6:**
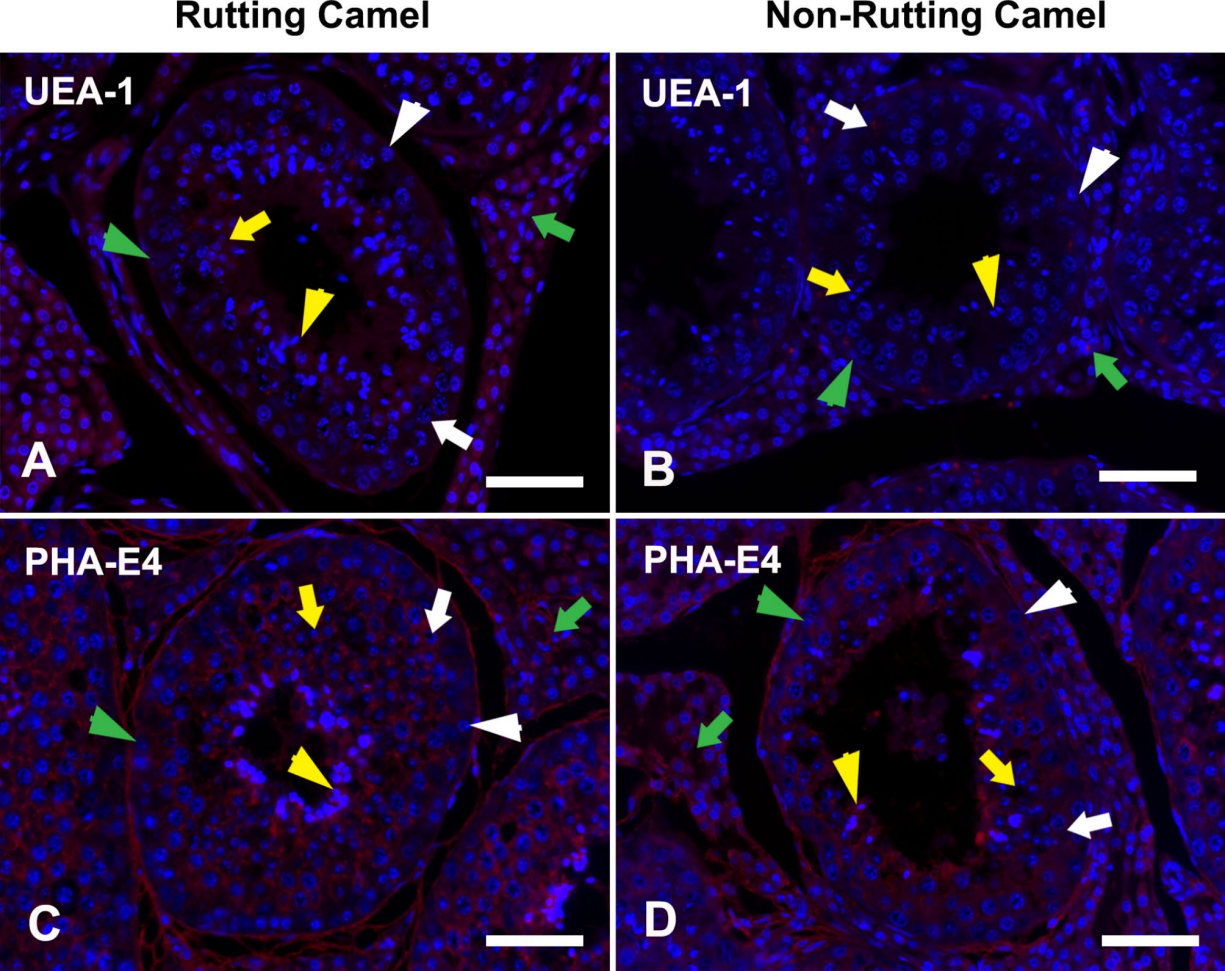
LHC in the testes of dromedary camel showing cellular localization of fucose binding lectin (UEA-1) and non-specific binding lectin (PHA-E4) during rutting **(A, C)** and non-rutting **(B, D)** seasons. The cellular nuclei were counterstained with Hoechst 33,258 (blue fluorescence). Spermatogonia; Sg: white arrowhead, Spermatocyte; Sp: white arrow, round spermatid; Rs: yellow arrow, elongated spermatid; Es: yellow arrowhead, Sertoli cell: green arrowhead, and interstitial cells; IC: green arrow. UEA-1 had a weak intensity in spermatocytes, and moderate intensity in spermatids and IC in rutting camel testes but in the non-rutting camel, it was absent except for weak labeling in spermatocytes and IC. PHA-E4 reacted to all testicular cells of both species except spermatogonia and Sertoli cells. Scale bar = 100 μm

UEA-1 had a weak intensity in spermatocytes, moderate intensity in spermatids, and IC in rutting camel testis. While in the non-rutting camel, it was absent except for weak labeling in spermatocytes and IC (Figs. 6A-B and 2E).

PHA-E4 represented the nonspecific binding lectin that was absent in spermatogonia and Sertoli cells while showing a strong reaction in the other testicular cells during both active and inactive seasons (Figs. 6C-D and 2E).

### Statistical analysis

The statistical analysis in Table 3 did not show any marked correlation between the response intensity and most of the indicator variables such as type and group of lectins and the cell type (p > 0.05). On the other hand, a marked correlation between the breeding season and response intensity was detected at P < 0.05. Odds ratio estimates showed a tendency for a non-significant stronger response depending on the name of the lectin used (OR: 1.176). Moreover, the intensity of response was modified by 0.994 and 0.751 points based on the cell type and lectin group, respectively. Breeding season significantly alters the intensity of response by 0.498 points.

**Table 3:** Results of generalized linear regression model for the association between the intensity of the response and different variables including the group of lectins, name of lectin, breeding season, and type of cell examined

Variable	SE	*P*- Value	Odd ratio	95% Confidence interval	
Lectin group	0.361	0.424	0.751	0.372	1.515
Name of Lectin	0.142	0.245	1.176	0.895	1.546
Breeding season	0.297	0.019*	0.498	0.278	0.891
Cell	0.086	0.947	0.994	0.843	1.173

*Indicates significant association between breeding season and intensity of response

## Discussion

Spermatogenesis in the camel is more active during the winter season, but it is reduced without complete spermatogenesis during the summer season [5, 10, 51]. Therefore, many factors affecting spermatogenesis showed variation between the two periods of the year. One of these factors is the distribution of different sugar residues in adult camel testicular cells during non-rutting and rutting seasons that have been investigated in the present study. Most of these residues are limited to the interstitial Leydig cells and germ cells, while the Sertoli cells are rarely reacted or even left unlabeled as previously reported by [3, 52].

The lectin histochemistry is attributed to revealing certain epitopes on the glycan chains of cellular glycoconjugates [30, 53, 54] that may give the sugar residues the ability to store biological data. Additionally, the importance of lectin histochemistry includes using lectin-specific markers in cytochemistry and histochemistry, purifying reactive sugar receptors, designing selective high-affinity ligands of glycoconjugates, and aiming to make long-term participation in structure-function relationships at the level of cells, molecules, organs, and tissues [53, 55].

The results of this study revealed that the camel testicular components reacted to the most investigated lectins (Glc, Gal, GlcNAc, GalNAc). In the camel testis, the structural allocation of the sugar moieties may suggest that specific sugar residues are crucial for spermatogenesis through different seasons. Therefore, their expression appeared in different patterns. The specific protein-carbohydrate interactions are considered an important factor responsible for controlling and regulating numerous regulatory processes, such as cell growth, apoptosis, glycoprotein folding and transport, and cell adhesion [53, 56]. All investigated lectins in the present study did not have any reactivity with Sertoli cells except SBA. This could be explained that the main role of sugar residues during spermatogenesis occurred through the spermatogenic cells [57] and interstitial cells, not the somatic cells.

The Glc-binding lectins (LCA and ConA) were restricted to Spermatogonia, Spermatocytes, and interstitial cells, particularly during the active breeding season in agreement with [3] where mannose and glucose residues are prevalent in the compounds having ion transport properties [58, 59]. Moreover, strong LCA reactivity in the basement membrane of seminiferous tubule was observed that may be necessary for interactions between Sertoli and germ cells during spermatogenesis [60].

The acrosomes of spermatids include many glycoproteins-associated enzymes such as acrosin, acid phosphatase, and hyaluronidase which are crucial for fertilization. Therefore, spermatids were the major lectin-linked cells in the camel testis [3, 61, 62]. Gal-binding lectins were mainly localized in the spermatid during both breeding seasons, particularly PNA that had no labeling with interstitial cells. Therefore, it could be used as an acrosomal marker during studying spermatogenesis by IHC. It is worth mentioning that the galactose residues were suggested as markers of cell differentiation [58, 63] and essential for intercellular adhesions [64, 65]. Furthermore, spermatid glycans engage in ion transport as well as contact and interaction with adjacent Sertoli cells [66]. Therefore, the galactose residues are common in glycans that interfere with fluid and ion transport, anchoring structure development and are also thought to be indicators of acrosomal differentiation [67, 68].

GlcNAc (WGA) had nearly the same labeling pattern in both rutting and non-rutting camels’ testis in Spermatogonia, Spermatocytes, round and elongated spermatids that may refer to its essential role during both seasons, particularly at the level of spermatogenic cells. On the other hand, previous investigations revealed that WGA was labeled in spermatocytes and spermatogonia only through the rutting season in addition to constant reactivity to the basal lamina of the seminiferous tubules and Leydig cells during both seasons [3].

Regarding GalNAc, there was a wide range of distribution in the seminiferous epithelium as well as interstitial cells by different intensities of response. The different reactivities within the same group of lectin may be due to various terminating oligosaccharides according to the variety of linkage and underlying glycoconjugate residues [69, 70] since similar monosaccharide binding by various lectins might result in different fine specificities.

The current study clarified that most investigated lectins were clearly reactive to the interstitial Leydig cells during both rutting and non-rutting seasons. It was previously reported that Leydig cells in the dromedary camel testis go through two phases of increased activity throughout the year. The first phase begins in the early months of winter, while the second, occurring in the summer, is not as obvious as the first one and may be necessary to keep spermatogenesis at a low level until the next highest activity period [71–73]. This indicates that camel Leydig cells are active throughout the year, with high activity during the active rutting period. This activity is similar to that of the spermatogenic epithelium.

The statistical analysis in the current study displayed a significant correlation between the response intensity and breeding season where the breeding season significantly alters the intensity of response by 0.498 points. These findings support the idea that a particular type of carbohydrate is necessary for effective spermatogenesis during the active breeding season [69].

In conclusion, the distribution profile of glycoconjugates within the camel testis is affected by breeding season and affects somatic-spermatogenic cellular adhesion which in turn affects spermatogenesis and reproductive performance. Furthermore, certain examined lectins particularly PNA and ECA were found to be restricted to the acrosomes of spermatids, which could be valuable for the investigation of various stages in the spermatogenic cycle of the dromedary camel.

## Data Availability

All data and materials were present in the article.
